# Comments on Occupation Tuberculosis

**Published:** 1917-08

**Authors:** Letson Balliet

**Affiliations:** Specialist in Efficiency Engineering


### COMMENTS ON OCCUPATIONAL TUBERCULOSIS.

### (Miner's Consumption.)

**By Letson Balliet, M.S., E.M., C.E., Specialist in Efficiency Engineering.**

It is with some reluctance that I undertake to write upon matters for the medical profession, for in some way, I got shunted out of my medical course while attending the university, and when I left, a lot of professors of the school signed a document about two feet square that they believed that I was an engineer instead of a doctor. As such my work for more than twenty years has been in the line of construction and reconstruction, and during my first two years in school with the "medicos" I got the idea that the doctors have something to do in the line of construction and reconstruction, so maybe we are not so far apart after all.

As a mining engineer I am perhaps a good deal like a "medico" for I have to stand outside a mountain and guess what are the conditions inside of it, and from the "symptoms" I must plan the equipment, development and treatment. So must the doctor stand outside the body and guess what the conditions inside may be, from the symptoms, and he too must plan the equipment, direct developments and treatments. The only differenct is in the final result. If I make a mistake I leave an inverted monument to marke the mistake, while the doctor's mistake is marked by an upright granite monument.

So the difference in our occupations isn't so great that

I haven't a right to make some guesses on your side of the fence, and if I mistake not some of you doctors who have bought mining stocks, have made guesses upon my side of the fence, and maybe—in some cases—its 50-50 on which side of the fence you made the highest per cent. of good guesses. Therefore, as I have stood for a whole lot of doctor's guesses in my life, you'll have to stand for one from me, on what I will call occupational tuberculosis.

For over twenty years I have been handling men in mines, quarries, excavations and concrete construction, and have lived surrounded by similar operations under other engineers. In the various mining operations from 25 per cent. to 45 per cent. of all deaths are assigned to tuberculosis and pneumonia,—pneumonia, no doubt, frequently fostered by lacerated and inflamed condition of the lungs, as hereafter mentioned.

Miners' consumption, or phthisis, is locally called "the con" or "the dust" and may or may not respond to any analysis for tubercular bacteria. Satisfactory remedies are nil, and so fatal is the malady in advanced stages that it makes little difference to the doctor, or the patient, whether it is "the dust" clogging the air cells of the lungs, or bacteria of tuberculosis.

It appears to me, that the dust from rock-drills and blasting, when breathed into the lungs by the workmen, may do one of two things, or both. First, the sharp edges of angular pieces, may lacerate the lung tissues, that will cause inflammation and swelling, and make the tissues more susceptible to infection by disease germs that may be present in the air of poorly ventilated mines, where fellow workmen are breathing the same air. Second, some dust becomes a pure mud with moisture, and such dust will simply clog every lung cell with mud, sealing them so tightly that the lung functions are prevented, even without a bacteria of any kind being present. The patient becomes anemic and wastes away with the symptoms of tuberculosis, and the death certificate is so made out. As I understand it, the tubercular germ of the real "white plague" exists in oxygen, and in itself is harmless, but it builds a colony, and multiplies rapidly, in the passage-ways and entrances to the cells, where it takes the oxygen out of the air, and thus prevents the blood from being aerated as it was intended to be by nature.

From this, it would seem that any "consumption cure,, to be successful would of necessity be a gas, that could be inhaled by the patient. This gas would have to kill the germs, without killing the patient, but it would be wholly incompetent to obtain results in cases where the dust had clogged the lung cells. How much of the "Miners' con" is tuberculosis, and how much is "the dust" I do not know, nor do I know of any systematic effort to ascertain. In both cases the symptoms and results are identical.

We all know that the body tries to fight its own diseases by manufacturing a toxin to combat the germs, and that it actually kills millions of them, and it is simply a question of whether the body kills them faster than they multiply, or whether they multiply faster that the body kills them. The open air remedy is the favorite among those who try to combat "miner's con" and tuberculosis, on the theory that the patient can inhale more oxygen than the tubercular colony can use. If this is successful the body builds up in strength, and kills the germs of the disease faster than they multiply, and recovery is possible, but the patient with the dust filled lungs gets no relief.

My intention in writing this article, is not to tell the medical profession anything about consumption cures, but to suggest in the name of humanity, that every doctor, the medical press, and those connected with the profession, bring every influence to bear to prevent the employment of tubercular subjects in the same place with healthy workers. The medical side of the argument I leave to you. As an efficiency engineer I have suggested this many times and met with opposition. In 1913 I suggested a law to require a medical examination before employment, and every sixty days during the period of employment. This would prevent infected men from being employed with healthy men, and also give the doctors a chance to discover early symptoms and thereby give the patient a chance for a cure. Then the storm broke around my head.

Laborers objected—"because it would put men out of employment who were not sick enough to be worried about it, and make them and their families starve."

Employers said, "what do we care. As long as a man is able to work, let him work and care for his family. If a dozen other men became infected, in our mine, we'll get all the work out of them that we want before they go, and others are easy to get." With migratory workers like miners there are times when there are "flocks" of men looking for work, and other times when the "flocks" have migrated and men are hard to get.

The taxpayers kicked because it would force a man who was not permitted to work to become a public charge, while there was yet a few months or years of work in him.

In answer to all these—it is better to have one man become a public charge, a year or two earlier than seems necessary, than to have ten or twenty that he has infected become public charges a few years later, and each of the subjects in the meantime having infected as many more.

Frequently the miners' bunkhouses and sleeping quarters are as crowded as a sleeping car (without the heat and ventilation of the sleeping car), and unkept, with more or less spitting by heedless men. These migratory workers will spread the disease until this becomes a tubercular nation, if steps are not soon taken to prevent it.

If necessary it is better to provide a tubercular home or farm, or colony, in every county, where they are provided with out-door life and work, than to let things continue till the nation becomes a tubercular home.

Healthy workers, certainly don't want to work with conditions that mean a certain death in a few years. They, above all others, should demand safety and protection. If employers realized that it costs money to break in new men, and that their employes of long experience do the best work, they certainly wouldn't want to kill off their experienced employes, by the time they have gained experience enough to be the most valuable, and thus deprive themselves of ten or fifteen or more years of the very best ability of the men they have trained.

Efficiency demands safety from accident and from disease. Humanity demands it, too. Every mining camp of any age has dozens of widows and orphans that would not be deprived of their support, had not the strong healthy man been infected with tuberculosis by fellow employes.

If similar conditions exist in some factories, sweat shops and tenements, the medical profession is in the best position to know of these cases where I as a mining engineer would have little opportunity to gain knowledge. There is much more to be said, about emigrants from southern Europe, who huddle together in crowded dwelling quarters, and migrate from place to place for work, but space is limited and if I have opened a subject for argument, or comment, the result may be beneficial. The more agitation the better for humanity.—The Medical Summary, Philadelphia, Pa.

